# Non-canonical Inflammasome-Mediated IL-1β Production by Primary Endometrial Epithelial and Stromal Fibroblast Cells Is NLRP3 and Caspase-4 Dependent

**DOI:** 10.3389/fimmu.2019.00102

**Published:** 2019-02-05

**Authors:** Paul Kelly, Kieran G. Meade, Cliona O'Farrelly

**Affiliations:** ^1^Comparative Immunology Group, School of Biochemistry and Immunology, Trinity Biomedical Sciences Institute, Trinity College Dublin, Dublin, Ireland; ^2^Animal and Bioscience Research Department, Animal and Grassland Research and Innovation Centre, Grange, Ireland; ^3^School of Medicine, Trinity College Dublin, Dublin, Ireland

**Keywords:** endometritis, inflammation, IL-1β, NLRP3 inflammasome, epithelial cells, stromal fibroblasts

## Abstract

Inflammation of the post-partum uterus is a normal physiological event, crucial for tissue involution and repair. However, in the bovine, some cows fail to resolve this inflammation, resulting in endometritis, which compromises fertility. Earlier work has identified upregulated expression of the potent inflammatory cytokine IL-1β early post-partum, in cows which subsequently develop endometritis. As a result, targeting IL-1β expression holds potential as a novel treatment for this disease, yet the regulatory mechanisms contributing to IL-1β expression in the bovine endometrium remain unknown. To investigate this, endometrial tissue samples were obtained 7 and 21 days post-partum (DPP) from cows that were diagnosed with endometritis at 21 DPP and cows that experienced a physiological level of inflammation throughout involution. IL-1β was measured by qPCR, ELISA, and immunohistochemistry. Seven DPP, endometrial IL-1β protein levels were significantly higher in animals that proceeded to develop endometritis at 21 DPP. IL-1β production could be detected in luminal and glandular epithelium, in underlying stromal fibroblasts as well as infiltrating immune cells. To investigate the mechanisms regulating IL-1β expression, primary endometrial epithelial cells, stromal fibroblasts and PBMCs were stimulated with LPS and the inflammasome activator nigericin. Stromal fibroblast cells were particularly potent producers of IL-1β. Basolateral LPS stimulation of polarized epithelial cells induced *IL1B* mRNA and a previously undescribed IL-1β protein isoform, with preferential protein secretion into the apical compartment. Key inflammasome components [nod-like receptor protein 3 (NLRP3), nima-related kinase-7 (NEK7), apoptosis speck like protein containing a CARD (ASC), and gasdermin-D] were expressed by endometrial cells following stimulation. Endometrial cell stimulation in the presence of NLRP3 receptor (MCC950) and pan-caspase (Z-VAD-FMK) inhibitors blocked IL-1β production, demonstrating its dependence on the NLRP3 inflammasome and on caspase activity. Furthermore, caspase-4 specific siRNA prevented IL-1β production, confirming that inflammasome activation in endometrial cells is caspase-4 but not caspase-1 dependent, as shown in other species. Identifying the tissue- and species-specificity of inflammasome assembly and activation has critical relevance for our understanding of inflammation and suggests new therapeutic targets to enhance the resolution of inflammatory pathologies including endometritis in cattle.

## Introduction

The uterus is an organ with a complex immune repertoire, required to maintain an immune-tolerant environment during the development of a semi-allogenic fetus while retaining its ability to detect and respond to infectious agents if required. Parturition is quickly followed by bacterial contamination of the uterus and tissue involution, resulting in a heightened inflammatory environment within the uterus post-partum, driven by local microbial associated molecular patterns (MAMPs) and damage associated molecular patterns (DAMPs). This has been described as a normal physiological inflammatory event, required for adequate tissue remodeling and restoration of homeostasis in the uterus in preparation for future conception (1). Within the bovine species, a significant proportion of cows fail to resolve this inflammation, resulting in prolonged inflammation associated with uterine pathology termed endometritis, which can present as subclinical disease (termed cytological endometritis) and/or as clinical disease [termed purulent vaginal discharge (PVD)]. Endometritis has negative consequences for cow health and fertility in addition to economic costs as a result of reduced milk yield and prolonged calving to conception rates (2, 3).

The molecular and physiological events surrounding the switch from a healthy inflammatory state in the uterus to that of a pathological phenotype remain poorly understood. The activation and production of a number of potent inflammatory cytokines have however been linked to disease development (4). Previous work by our group has demonstrated that IL-1 family members are differentially expressed between healthy and endometritic cows as early as day 7 post-partum, as evaluated by RNA-seq and qPCR on endometrial tissue biopsies (4). Additionally, IL-1β levels were also recently shown to be significantly higher in cervical-vaginal mucus samples from cows with endometritis when compared to healthy cows 7 and 21 DPP (5).

The IL-1 family includes the cytokines IL-1β, IL-1α, IL-18, and IL-33, among others (6). Members of the IL-1 family and in particular IL-1β have been implicated in several inflammatory pathologies in cattle. IL-1β expression is raised in cattle infected with *Mycobacterium avium* subspecies *paratuberculosis*, the causative agent of Johne's disease (7). Similarly, a role for IL-1β has been associated with resistance to *Mycobacterium tuberculosis* infection and with the pathogenesis of bovine mastitis (8, 9). With regard to uterine disease, low levels of IL-1α expression has been documented in endometrial cells in response to tissue damage (10). However, the role of IL-1β in post-partum uterine pathology remains unexplored.

Interleukin-1β is produced in an inactive pro-form that requires cleavage for release from the cell and subsequent biological activity. An associated complex of proteins known as the inflammasome is responsible for mediating this cleavage (11). A number of different inflammasome complexes have been described, with the best characterized being nod-like receptor protein 3 (NLRP3). The NLRP3 inflammasome complex is activated by several diverse stimuli including the microbial toxin nigericin, DAMPs such as ATP and the commonly used vaccine adjuvant alum (12, 13). Inflammasome activation requires two signals, the first is usually a pathogen associated molecular pattern (PAMP) such as LPS which induces expression of pro-IL-1β; the second signal causes activation and oligomerization of the NLRP3 receptor which in turn recruits the adaptor protein apoptosis associated speck-like protein containing a CARD (ASC). ASC then mediates cleavage of pro-caspase-1 into its active form, allowing it to cleave IL-1β into its active form (11, 14).

While inflammasome complex formation and IL-1β production have classically been associated with immune cells such as macrophages and dendritic cells, their role in cells that comprise endometrial tissue such as epithelial cells and underlying stromal fibroblasts remains unclear (15). Given the evidence of IL-1β association with inflammatory disease, we hypothesized that local inflammasome activation in the endometrium and subsequent release of the pro-inflammatory cytokine IL-1β might play a role in the inflammatory response associated with post-partum endometritis in dairy cows. Here, we examine healthy and endometritic tissue for IL-1β expression, investigate the production of IL-1β by endometrial epithelial cells and stromal fibroblasts and explore the inflammasome pathways regulating IL-1β production in these cell populations.

## Results

### IL-1β Levels Are Higher in Endometrial Tissue in Cows That Develop Endometritis at Both 7 and 21 DPP

**Figure 1:**
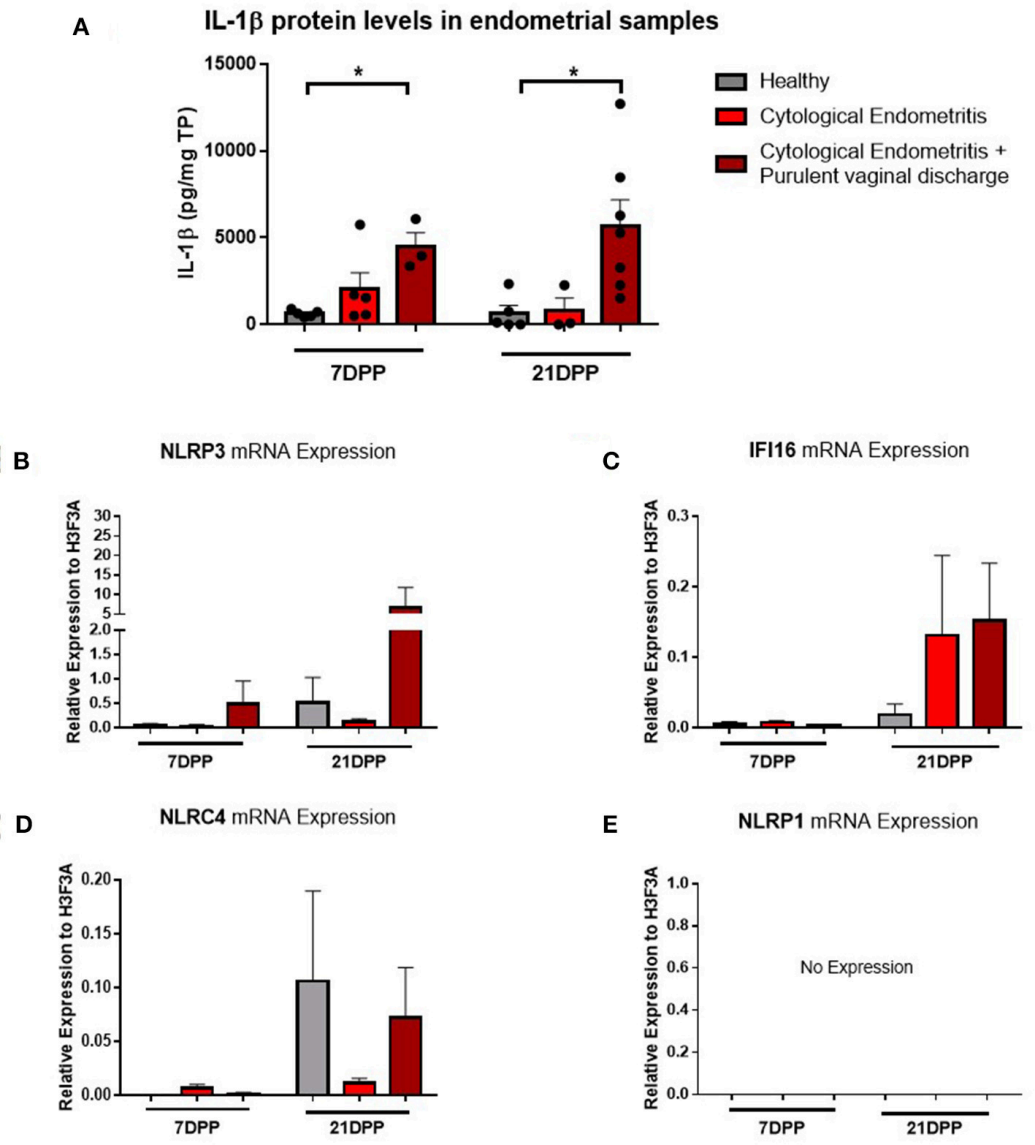
IL-1β protein levels and NLRP3 receptor mRNA expression are elevated in endometritic animals. Protein and RNA were extracted from endometrial samples obtained by cytobrush at 7 and 21 DPP from healthy cows and cows with cytological endometritis alone or cytological endometritis with PVD. **(A)**. Protein levels were quantified via BCA assay and IL-1β levels quantified by a bovine specific ELISA kit. Data is presented as mean cytokine expression (pg) per mg of total protein + SEM (*n* = 3–7). ^*^P < 0.05 was calculated using a Mann Whitney test in GraphPad Prism 7 software. **(B–E)** NLRP3, *IFI16, NLRC4*, and *NLRP1* inflammasome receptor expression were quantified via qPCR on mRNA extracted and reverse transcribed to cDNA. Data are presented as mean mRNA expression + SEM relative to the reference gene *H3F3A* (*n* = 3–7).

Endometrium from healthy and endometritic cows were sampled at two time points post-partum (7 and 21 DPP) using endometrial cytobrushes from which RNA and protein were extracted. IL-1β protein levels were quantified by ELISA and found to be significantly elevated in animals diagnosed with cytological endometritis and PVD at both time points (Figure 1A).

Using the RNA extracted from these endometrial samples we examined expression of the main inflammasome receptors (NLRP3, IFI16, NLRC4, and NLRP1). NLRP3 demonstrated the highest level of expression (Figure 1B) and so the NLRP3 inflammasome became the focus of our subsequent investigations into the IL-1β regulation in the endometrium. Low expression levels of the genes encoding the inflammasome receptors IFI16 and NLRC4 were detected but no expression of *NLRP1* (Figures 1C–E). The AIM2 inflammasome receptor has previously been reported to be pseudogenised in the bovine rendering it non-functional and so its expression was not examined here (16).

**Figure 2:**
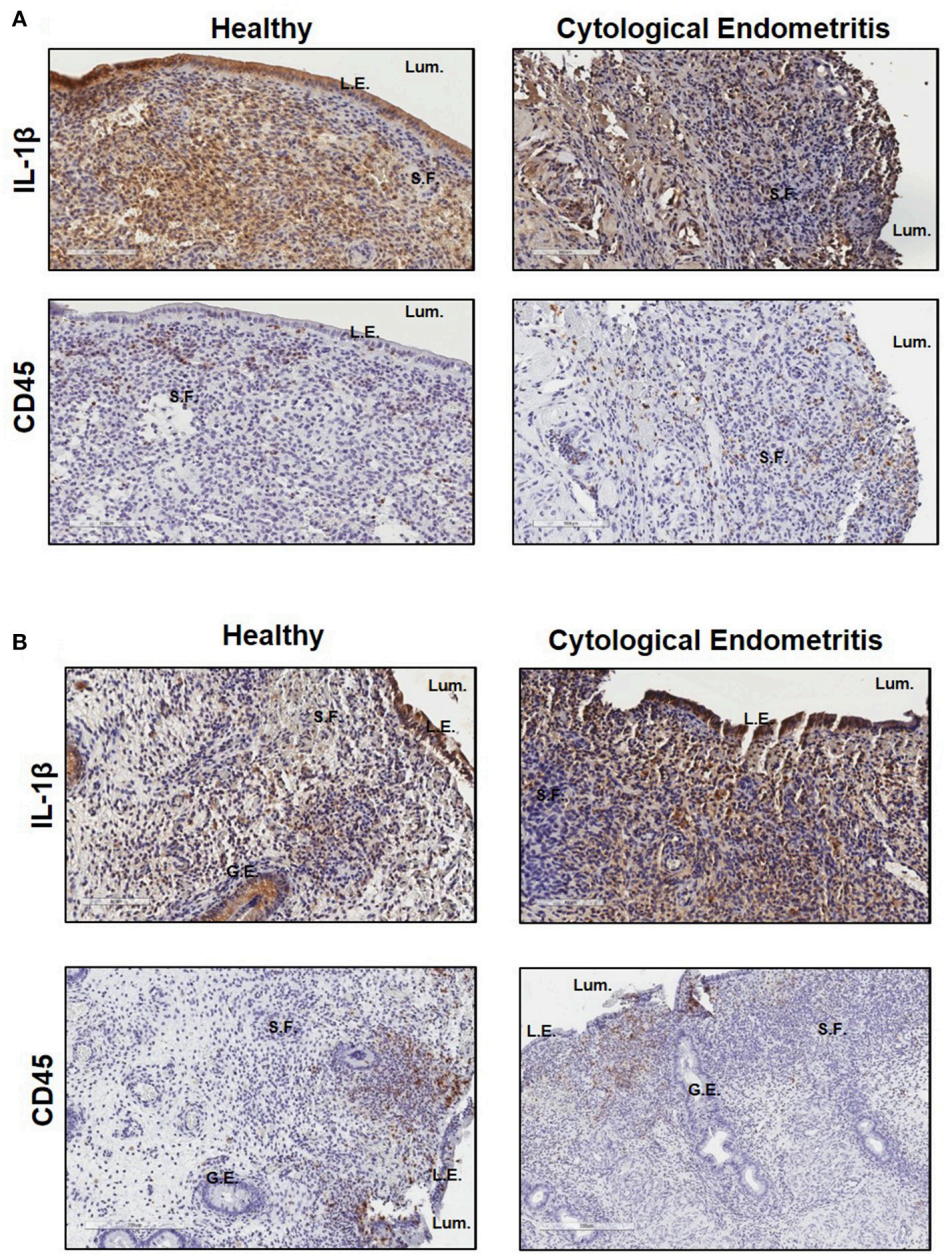
IL-1β protein is primarily localized to the epithelial cells and stromal fibroblasts in the post-partum endometrium. Endometrial biopsies were collected from healthy or endometritic cows at either **(A)** 7 DPP or **(B)** 21 DPP. Cows were classified as healthy or as having cytological endometritis based on immune cell infiltration at 21 DPP. Biopsies were paraffin embedded and subsequently sectioned and stained for IL-1β or CD45. Nuclei were counterstained with haemotoxylin. Tissue sections were visualized using the Aperio ScanScope Imager under 20x magnification. Scale bar indicates 100 μm. Lum., uterine lumen; L.E., luminal epithelium; G.E., glandular epithelium; S.F., stromal fibroblasts.

Immunohistochemistry was subsequently used to localize IL-1β expression within endometrial biopsies. Strong staining for IL-1β was visible in sections from both healthy and endometritic animals 7 DPP. CD45 staining localized immune cells to the upper functional layer of the endometrium. Comparing IL-1β and CD45 staining, it is evident that IL-1β production is not localized exclusively to infiltrating immune cells; strong expression is also evident in in the luminal and glandular epithelial cells and underlying stromal fibroblasts (Figure 2A). By 21 DPP IL-1β expression in healthy animals had begun to resolve while strong expression of IL-1β was still evident in biopsy sections taken from cows diagnosed with uterine disease (Figure 2B).

### Stromal Fibroblasts and Polarized Epithelial Cells Are Potent Producers of IL-1β

Given the high level of IL-1β expression observed in epithelial and stromal fibroblast cells by immunohistochemistry, we aimed to elucidate the contribution of these cell types to IL-1β mediated inflammation in the endometrium.

**Figure 3:**
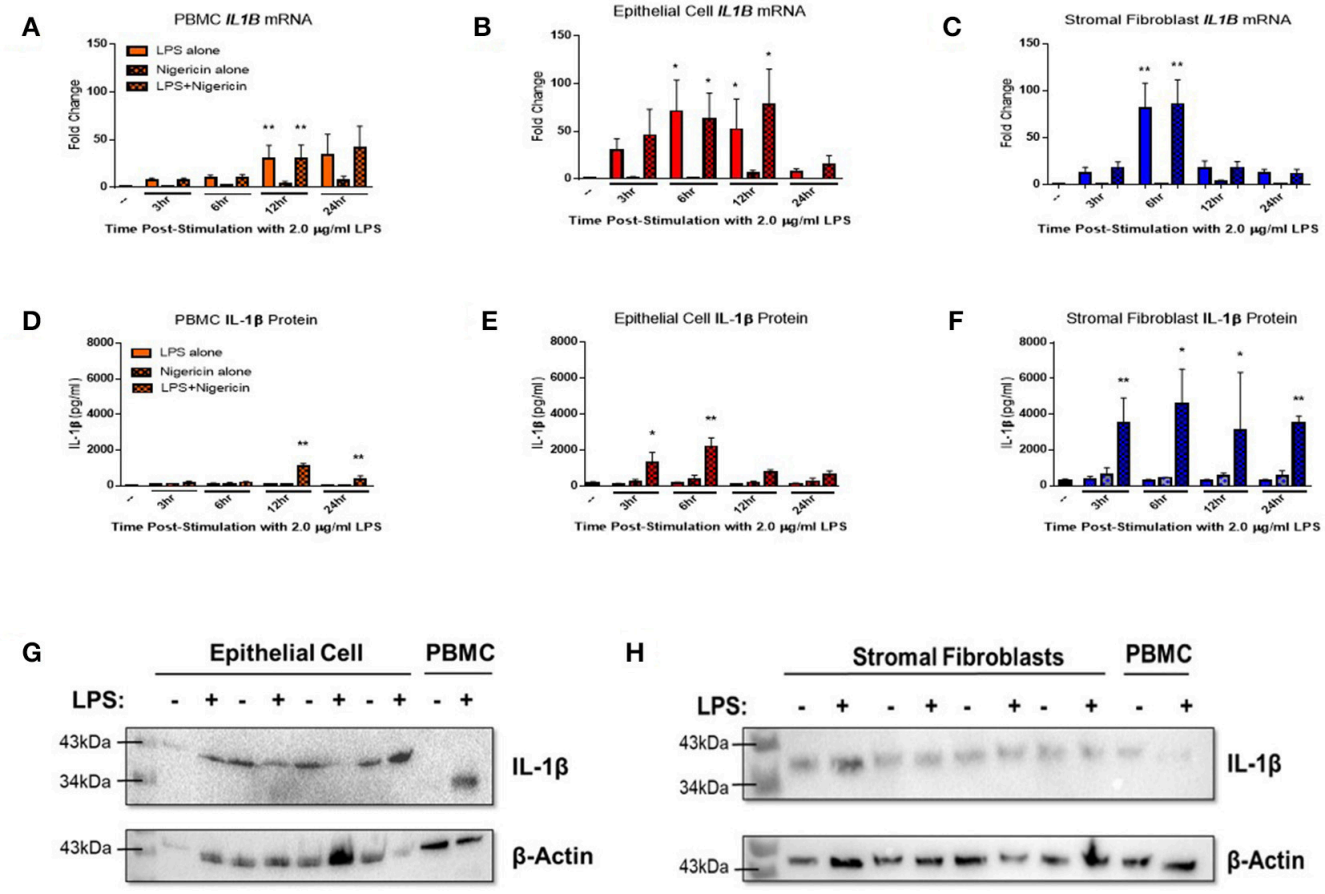
IL-1β production is induced in endometrial cells in response to LPS and the inflammasome activating signal nigericin. Expression of IL-1β was examined in primary endometrial epithelial, stromal fibroblasts, and peripheral blood mononuclear cells in response to stimulation with LPS and the inflammasome activator nigericin over a time course of 24 h. Expression of *IL1B* mRNA **(A–C)** and IL-1β protein production **(D–F)** in PBMCs, epithelial cells and stromal fibroblasts following stimulation with 2 μg/ml LPS over a time-course of 24 h and subsequent stimulation with 10 μM nigericin for 1 h. Induction of mRNA was examined by qPCR and protein production was examined by a bovine specific IL-1β ELISA in cultured supernatants (*n* = 5). Pro-IL-1β protein expression was examined in epithelial cells and stromal fibroblasts by western blotting **(G,H)**. For qPCR analysis, results are presented as mean fold change (+SEM) relative to the reference gene *H3F3A*. For ELISA analysis results are presented as mean cytokine concentrations (+SEM) in cell supernatants. ^*^p < 0.05, ^**^p < 0.01 were calculated using a Mann Whitney test in GraphPad Prism 7, comparing untreated control to a timepoint stimulation.

Endometrial epithelial and stromal fibroblasts were stimulated over a time course of 24 h with LPS as the inflammasome priming signal, followed by stimulation with the inflammasome activating ligand nigericin for 1 h. Endometrial cell responses were compared to the more traditionally studied immune cells from unmatched peripheral blood. Epithelial and stromal fibroblast cells demonstrated strong induction of *IL1B* mRNA in response to the inflammasome priming signal LPS alone or in combination with nigericin compared to PBMCs (Figures 3A–C). Induction of *IL1B* mRNA was highest in stromal fibroblasts 6 h post-stimulation with LPS.

Stimulation with both LPS and nigericin is required for IL-1β protein secretion across all three cell populations. Stromal fibroblasts produced most IL-1β, with production sustained over the 24 h time period (Figure 3F). IL-1β production by epithelial cells peaked significantly at the earlier time points of 3 and 6 h post-stimulation, while in PBMCs IL-1β expression peaked later at 12 and 24 h post-stimulation (Figures 3D,E). Interestingly, epithelial cell lysates probed for IL-1β protein expression by Western blotting (Figure 3G and Supplemental Figure 1) were found to produce a higher molecular weight protein (~43 kDa) than is traditionally observed in PBMCs (35 kDa) or by stromal fibroblasts (Figure 3H and Supplemental Figure 2).

**Figure 4:**
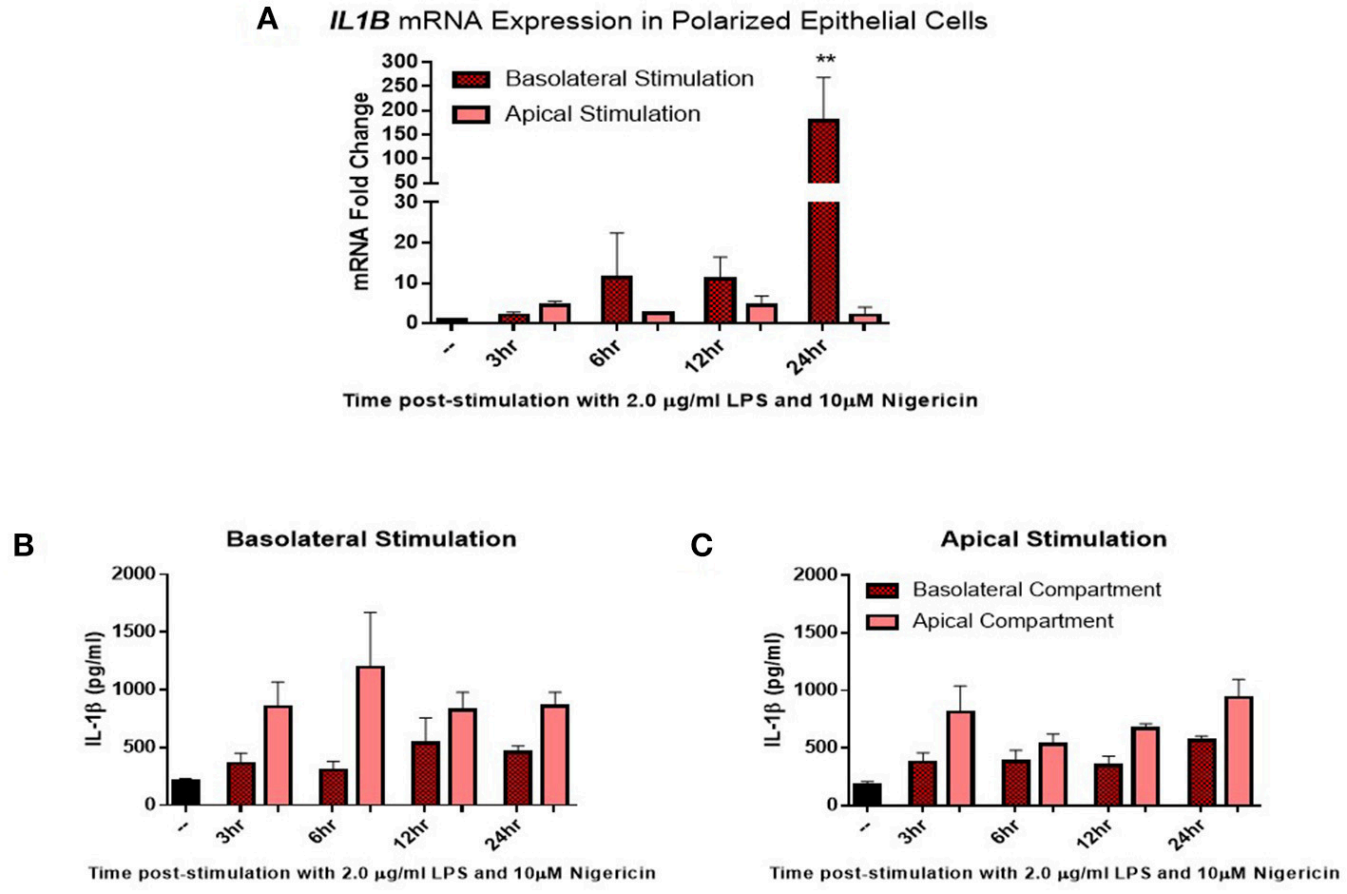
IL-1β induction in polarized epithelial cells is mediated via the basolateral membrane. Expression of *IL1B* mRNA **(A)** and IL-1β protein secretion **(B,C)** by polarized endometrial epithelial following basolateral or apical stimulation with LPS (2 μg/ml) and nigericin (10 μM) over a time course of 24 h (*n* = 5). For qPCR analysis, results are presented as mean fold change (+SEM) relative to the reference gene *H3F3A*. All values were normalized to the untreated control within the same time point. For ELISA analysis results are presented as mean cytokine concentrations (+SEM) in cell supernatants. ^**^p < 0.05 was calculated using a Mann Whitney test in GraphPad Prism 7, comparing untreated control to a timepoint stimulation.

Given the distinct 3D structure and orientation possessed by epithelial cells *in-vivo*, their responses *in-vitro* may be limited by their 2D culture. To investigate this, epithelial cells were grown on transwell inserts coated with matrigel and allowed to polarize for 14 days before stimulation. Confluency was measured using trans-epithelial resistance (TER). TER is the measurement of electrical resistance across a cellular monolayer and is a very sensitive and reliable method to confirm the integrity and permeability of the monolayer. Upon polarization, epithelial cells were stimulated with LPS (2 μg/ml) and nigericin (10 μM) either apically (representing stimulation from the uterine lumen) or basolaterally (representing a breach in the epithelial barrier and stimulation from the stroma compartment) (Figure 4). Significantly higher levels of *IL1B* mRNA induction were observed when stimulated from the basolateral compartment at 24 h post-stimulation in comparison with stimulation from the apical compartment (Figure 4A). We next examined whether this mRNA induction translated to protein production and whether protein production was directed toward a specific compartment. Both basolateral and apical stimulation with LPS and nigericin resulted in IL-1β protein accumulating within the apical compartment (Figures 4B,C).

### Endometrial Epithelial and Stromal Fibroblasts Produce the Inflammasome Components Required for IL-1β Secretion

Given the limited data on inflammasome signaling pathways within endometrial cells and particularly in the bovine, we explored expression of the relevant NLRP3 inflammasome components in endometrial cells compared to the classical IL-1β producing circulating immune cells. PBMCs, endometrial epithelial cells, polarized epithelial cells and stromal fibroblasts were all stimulated with the inflammasome priming PAMP LPS over a time course of 24 h followed by stimulation with the inflammasome activator nigericin for 1 h.

**Figure 5:**
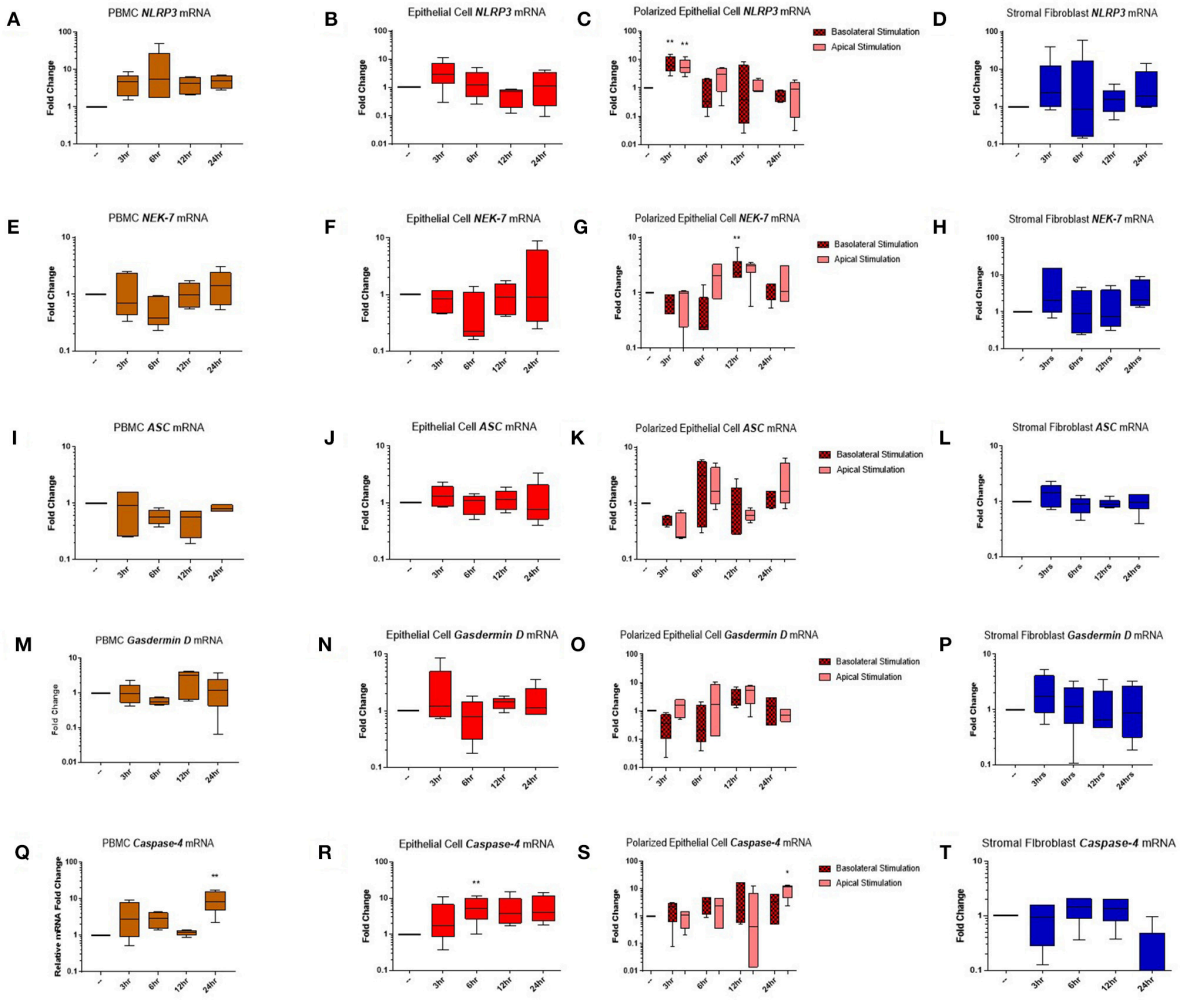
Inflammasome components are expressed in endometrial cells in response to inflammatory stimuli. Expression of components of the NLRP3 inflammasome complex were examined in PBMCs and primary endometrial epithelial cells and stromal fibroblasts following stimulation with LPS and the inflammasome activator nigericin over a time course of 24 h. NLRP3 **(A–D)**, NEK-7 **(E–H)**, ASC **(I–L)**, gasdermin-D **(M–P)**, and *caspase-4* **(Q–T)** mRNA expression was examined across PBMCs, 2D cultured epithelial cells, polarized epithelial cells and stromal fibroblasts (*n* = 5). Results are presented as mean fold change (+SEM) relative to the reference gene *H3F3A*. All values were normalized to the untreated control within the same time point. ^*^p < 0.05, ^**^p < 0.01 were calculated using a Mann Whitney test in GraphPad Prism 7, comparing untreated control to a timepoint stimulation.

Genes encoding the inflammasome components NLRP3 (Figures 5A–D), NEK-7 (Figures 5E–H), ASC (Figures 5I–L), gasdermin D (Figures 5M–P), and caspase-4 (Figures 5Q–T) were detected across PBMC, epithelial cell, polarized epithelial cell and stromal fibroblast stimulations. NLRP3 expression was significantly elevated in polarized epithelial cells 3 h following stimulation, although expression was not significantly different between apical or basolateral stimulation (Figure 5C). NEK-7 expression peaked significantly in polarized epithelial cells following 12 h of basolateral stimulation (Figure 5G). *Caspase-4* expression was significantly elevated in epithelial cells at 6 h post-stimulation (Figure 5R) and in PBMCs and apically stimulated polarized epithelial cells following 24 h of stimulation (Figures 5Q,S).

### Endometrial Epithelial Cells and Stromal Fibroblasts Are Dependent on the NLRP3 Receptor and Caspase Activity for IL-1β Secretion

Given the evidence presented here supporting a role for epithelial and stromal fibroblasts in mediating IL-1β production in the endometrium, we aimed to determine if IL-1β production was dependent on the inflammasome complex for its release using inhibitors targeting specific inflammasome components. The recent development of a small molecule inhibitor specific to the NLRP3 receptor has proven to be effective in both human and mouse studies (17). Here, we aimed to utilize this to investigate the contribution of the NLRP3 inflammasome receptor to IL-1β secretion in the endometrium.

**Figure 6:**
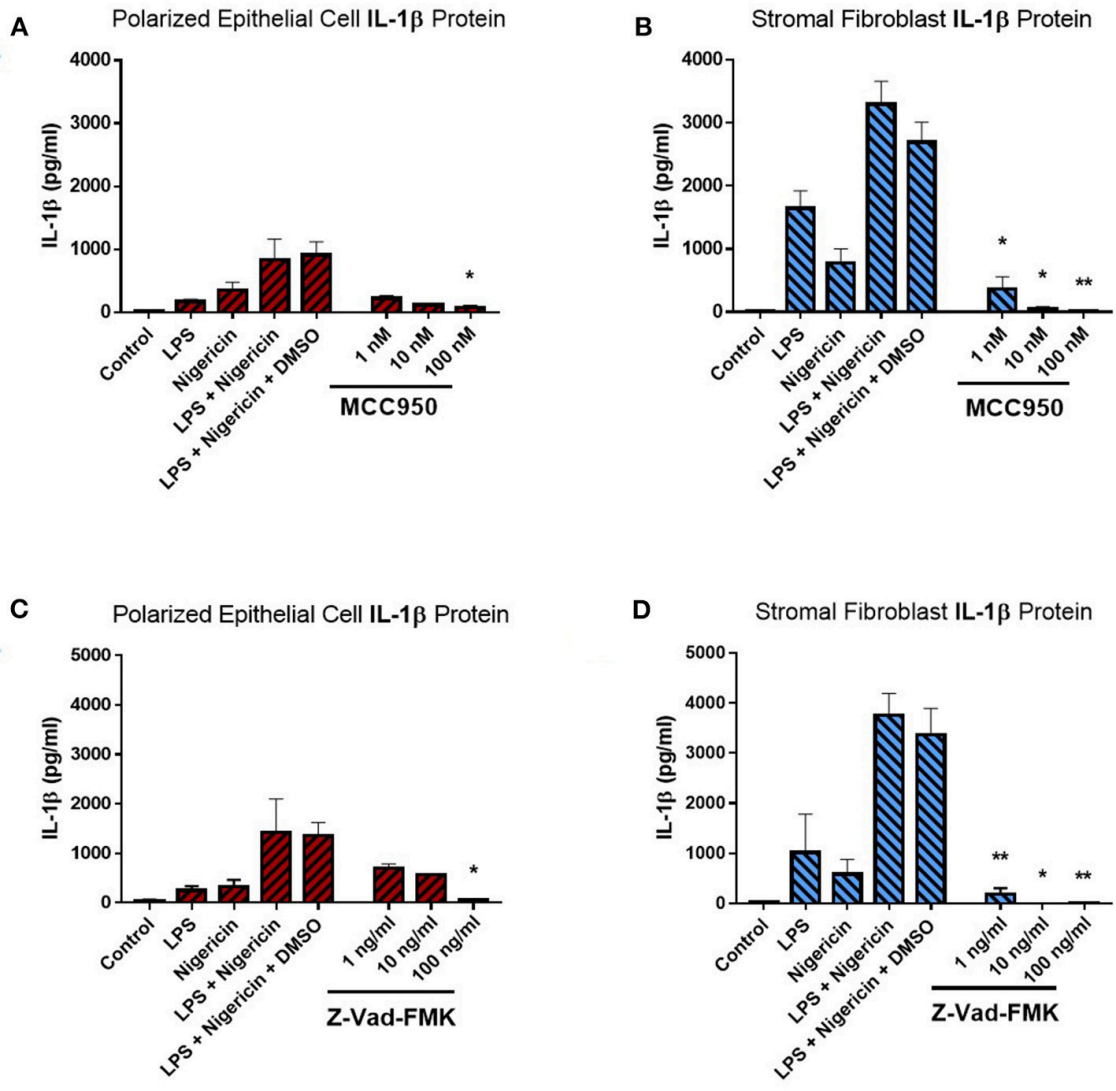
IL-1β production in endometrial cells is dependent on the NLRP3 inflammasome complex. IL-1β secreted from polarized endometrial epithelial cells and stromal fibroblasts was examined in the presence of inhibitors targeting specific components of the NLRP3 inflammasome complex. Cells were either treated with increasing concentrations of **(A,B)** the NLRP3 receptor inhibitor MCC950 (1–100 nM) before LPS priming or **(C,D)** the pan-caspase inhibitor Z-VAD-FMK (1–100 ng/ml). Treatment with DMSO was included as a vehicle control. Supernatants were subsequently harvested and IL-1β measured using a bovine specific ELISA (*n* = 5). Results are mean cytokine concentrations (+SEM). ^*^p < 0.05, ^**^p < 0.01 were calculated using a Mann Whitney test in GraphPad Prism 7, comparing the LPS and nigericin stimulation to individual inhibitor treatments.

Treatment of endometrial epithelial cells and stromal fibroblast cells with increasing concentrations of MCC950 prior to LPS priming and nigericin treatment resulted in marked decreases of IL-1β secretion from both cell populations (Figures 6A,B). Stromal fibroblasts appeared more sensitive to MCC950 than the epithelial cells which required higher concentrations of the inhibitor in order to achieve a significant reduction in the levels of IL-1β secretion. Treatment with MCC950 had no effect on epithelial cell viability (as quantified by measuring TER) or stromal fibroblast viability (Supplemental Figures 3A,B). MCC950 also had no effect on IL-8 cytokine production by either cell population, indicating its inhibitory effects are specific to IL-1β production (Supplemental Figures 3C,D).

We next aimed to determine whether IL-1β production was dependent on caspase activity using the pan-caspase inhibitor Z-VAD-FMK. Treatment with increasing concentrations of Z-VAD-FMK resulted in marked reduction of IL-1β production by epithelial and stromal fibroblast cells, with the stromal fibroblasts appearing more sensitive to the inhibitor than epithelial cells, as had been observed with MCC950 treatment (Figures 6C,D). Treatment with Z-VAD-FMK had no effect on cell viability or epithelial resistance properties and did not affect production of the chemokine IL-8, indicating that its effects are specific to IL-1β inhibition (Supplemental Figures 3E–H). However, given the role caspase enzymes play in programmed cell death, inhibiting their effects *in-vivo* may have implications for the processes of uterine involution and tissue remodeling post-partum. Therefore, a more targeted approach to inhibiting caspase activity would be preferred.

### IL-1β Activation in Endometrial Cells Is Dependent on Caspase-4

Canonical NLRP3 inflammasome pathway activation, as documented in human and murine studies, typically requires caspase-1. Previous work in bovine PBMCs has demonstrated caspase-1 expression and identified its role in mediating IL-1β production (18). However our previous RNA-seq experiments showed no expression of caspase-1 in endometrial biopsies whether from either healthy or diseased animals, while caspase-4, among a number of other inflammatory caspases, was found to be differentially expressed 21 DPP between healthy and endometritic cows (4). Caspase-4 has previously been reported as caspase-13, a novel bovine caspase, however subsequent bioinformatic analysis has revealed the sequence of capase-13 to be identical to caspase-4 (19). Bovine caspase-4 is the ortholog of caspases-4 and 5 in the human and caspase-11 in the mouse.

**Figure 7:**
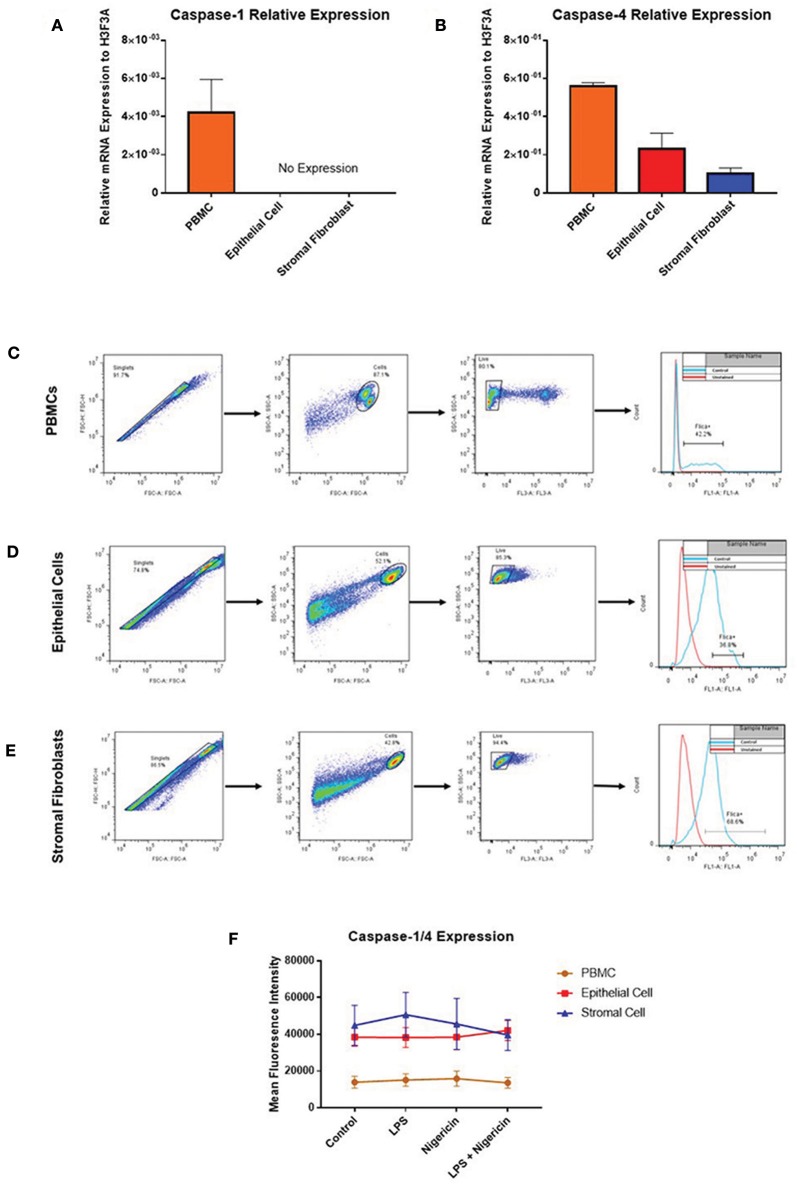
Endometrial cells display differential expression of inflammatory caspases. Levels of caspase-1 and caspase-4 expression was examined in PBMCs, endometrial epithelial cells and stromal fibroblasts (*n* = 5). **(A,B)** mRNA expression of the inflammatory caspases *caspase-1* and *caspase-4* was examined in PBMCs and primary endometrial epithelial cells and stromal fibroblasts via qPCR. Expression levels are relative to the reference gene *H3F3A*. Expression of active levels of caspase-1/caspase-4 in was examined by flow cytometry using the FAM-FLICA® caspase assay following stimulation with the inflammasome activators LPS (2 μg/ml) for 6 h and subsequent stimulation with nigericin for 1 h. Gating strategies for PBMCs **(C)**, epithelial cells **(D)**, and stromal fibroblasts **(E)** are shown. **(F)** Expression levels of caspase-1/caspase-4 in PBMCs, epithelial cells and stromal fibroblasts.

While profiling inflammasome component expression within stimulated endometrial cells, we found we were unable to detect expression of caspase-1 at the mRNA level, despite detecting caspase-1 expression in PBMCs (Figure 7A). Caspase-4 expression was detected across all three cell populations (Figure 7B) and shown to be inducible in response to inflammatory stimuli in PBMCs and epithelial cells but not in stromal fibroblasts (Figures 5Q–T).

The lack of commercially available species-specific antibodies compromises studies of protein expression of these inflammatory caspases within the bovine. The FAM FLICA® flow cytometry based assay had previously been shown to cross react with bovine caspases. However, this assay is limited by its inability to discriminate between caspase-1 and caspase-4, giving combined reports on active caspase-1 and caspase-4 activity. PBMCs, endometrial epithelial cells and stromal fibroblasts were primed with LPS for 3 h followed by stimulation with the inflammasome activator nigericin for 1 h. Cells were then treated with the caspase detection probe FLICA 660-YVAD-FMK for 30 min. The probe is cell permeable and binds irreversibly to active caspase-1 or caspase-4 present in cells. Results can then be quantified by flow cytometry, gating on single, live cells, positive for the FLICA probe (Figures 7C–E). Mean fluorescence intensity of the cells staining positive for caspase-1/4 was examined. We observed highest caspase activity levels in stromal fibroblasts and epithelial cells, which is consistent with the high levels of IL-1β production we observed in these cell populations (Figure 7F). Levels of caspase activity in PBMCs are consistent with previous observations in bovine PBMCs [(18); Figure 7F].

**Figure 8:**
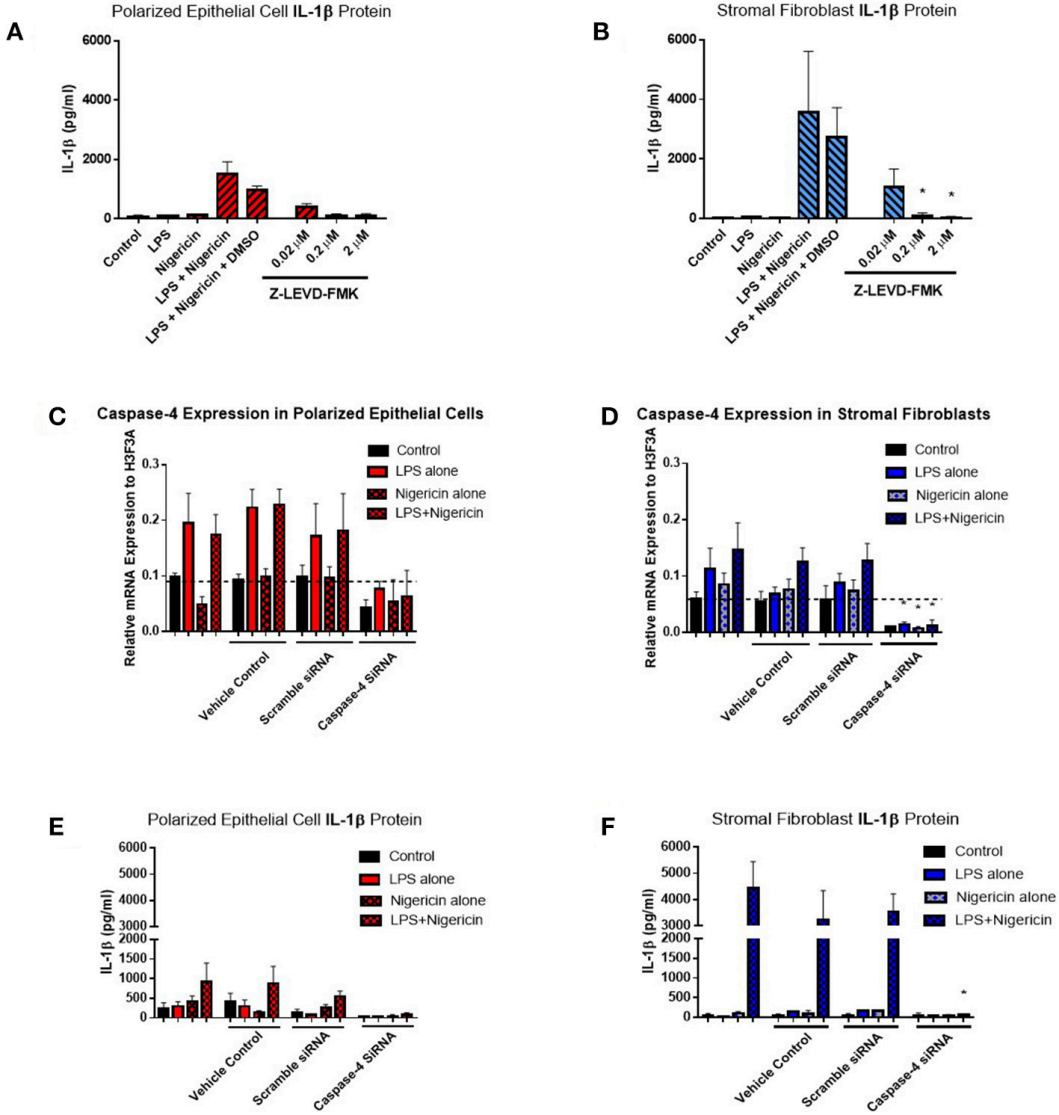
Inflammasome activation of IL-1β is mediated through caspase-4 in endometrial cells. IL-1β secreted from polarized endometrial epithelial cells and stromal fibroblasts was examined in the presence of a commercial inhibitor or siRNA targeting caspase-4 (*n* = 5). **(A,B)** Cells were treated with increasing concentrations of the caspase-4 inhibitor Z-LEVD-FMK (0.02–2 μM) before LPS priming. Supernatants were subsequently harvested and IL-1β measured using a bovine specific ELISA. **(C,D)** Caspase-4 mRNA expression levels in epithelial and stromal cells was examined by qPCR and **(E,F)** IL-1β measured by ELISA following treatment with siRNA directed against caspase-4, scrambled siRNA, a vehicle control or left untreated for 48 h followed by stimulation with LPS and nigericin for 6 h. mRNA expression levels are relative to the reference gene *H3F3A*. ELISA results are mean cytokine concentrations (+SEM). ^*^p < 0.05, were calculated using a Mann Whitney test in GraphPad Prism 7, comparing the LPS and nigericin stimulation to individual inhibitor treatments.

Given the difficulties in elucidating caspase-4 expression at the protein level, we aimed to examine its contribution to IL-1β production by inhibiting its activity through the use of a commercially available inhibitor and siRNA directed against caspase-4. Use of increasing concentrations of the commercial caspase-4 inhibitor Z-LEVD-FMK reduced IL-1β levels to levels observed in the control samples and significantly reduced IL-1β production by stromal fibroblasts (Figures 8A,B). Use of Z-LEVD-FMK had no effect on cell viability or IL-8 chemokine production (Supplemental Figure 3). Given the lack of specificity observed with some commercial caspase inhibitors, we wanted to validate our observations with the commercial caspase inhibitor using siRNA specific to caspase-4. Treatment of epithelial cells or stromal fibroblasts with caspase-4 siRNA reduced the levels of capase-4 mRNA present in both cell populations (Figures 8C,D). Treatment of both cell populations with scrambled siRNA or with the vehicle control alone had no effect on caspase-4 expression levels indicating that the siRNA is specific in knocking down its target. This knockdown of caspase-4 completely abrogated IL-1β production in both epithelial cell and stromal fibroblasts, while scrambled siRNA and vehicle control alone had no effect (Figures 8E,F). Additionally, treatment with caspase-4 siRNA had no effect on cell viability or IL-8 chemokine production (Supplemental Figure 4).

## Discussion

Prolonged or dysregulated inflammation is known to cause the pathology underpinning bovine endometritis (20). There is an urgent need to understand the role inflammatory cytokines play in initiating and propagating this pathological inflammatory response, particularly in non-model species. The IL-1 family of cytokines exhibits potent pro-inflammatory properties at sites of tissue infection or damage, acting on immune cells to drive their function and influence their survival. Limited expression data are available on IL-1 family members in the bovine species. Endometrial cell populations have been reported to produce small amounts of IL-1α in response to tissue damage (10). A role for the cytokine IL-1β has been widely studied in mouse and human models of inflammatory disease, with evidence of its role in bovine uterine disease emerging in recent years (4, 5). Additionally, macrophages from the high yielding Holstein-Friesian dairy breed have been found to produce significantly higher levels of IL-1β than the Brown-Swiss beef breed which may contribute to breed-specific differences in disease susceptibility (21). Given the potency of this cytokine, production of IL-1β at the protein level is a tightly regulated process controlled by the inflammasome complex. As differences in inflammasome signaling across cell types and species has begun to emerge in recent years (22–24), this study aimed to establish how the inflammasome was regulating IL-1β production within the bovine endometrium.

Earlier work has identified elevated gene expression of *IL1B* in endometrial biopsies obtained from cows diagnosed with post-partum endometritis (4), but our study is the first to confirm elevated levels of IL-1β protein in endometrial samples. Localizing this IL-1β production by immunohistochemistry to the epithelium and stromal fibroblasts demonstrates the predominant local production of this cytokine by the innate cells of the endometrium, as opposed to invading immune cells. Our immunohistochemical analysis also revealed more disruption to the epithelium in the endometritic cows, resulting in the underlying stroma being exposed. Recently, a role of fibroblasts in mediating inflammatory responses has been recognized, with fibroblasts implicated in maintaining chronic inflammation (25). Limited work has been performed in livestock models although bovine epithelial cells and stromal fibroblasts have been shown to produce IL-8 and IL-6 in response to inflammatory stimuli (26–28). We proposed that exposure of the epithelium and underlying stromal fibroblasts to the uterine microflora leads to the amplification of both epithelial cell and stromal fibroblast IL-1β expression, resulting in luminal leakage of this potent cytokine, which we have recently successfully detected in vaginal mucus (5).

In this study we have found that both polarized epithelial cells and stromal fibroblasts are potent producers of IL-1β when stimulated with LPS and the microbial toxin nigericin. The ability of epithelial cells, and inability of stromal fibroblasts, to directionally secrete IL-1β has several implications for the cytokine's role in uterine disease. Directing secreted IL-1β into the uterine lumen ensures that the cytokine is localized to the primary site of infection, ready to activate neutrophils recruited to the lumen. In contrast, the undirected release of IL-1β by stromal fibroblasts has the potential to propagate the inflammatory response within the endometrial tissue. IL-1β has previously been shown to affect intestinal epithelial tight junctions, resulting in permeability of the epithelial barrier (29). Thus, the production of IL-1β by stromal fibroblasts has the potential to limit the ability of epithelial cells to regulate inflammation which could ultimately lead to pathology.

Analysis of IL-1β production by Western blotting highlighted a number of key issues. As observed previously in bovine PBMCs (18), unstimulated endometrial epithelial cells and stromal fibroblasts contain pre-formed IL-1β which can be released from the cell in response to nigericin alone. Additionally, we describe a higher molecular weight isoform of IL-1β in epithelial cells in this study. We investigated a number of possible transcriptional and post-translational modifications that may account for the higher molecular weight of this cytokine isoform but were unable to identify the mechanism responsible. Within the literature, only one study exists reporting a higher molecular weight form of IL-1β found in human amniotic fluid (30). Given the directional secretion of IL-1β observed in our polarized epithelial cells, it is possible that the higher molecular weight isoform of IL-1β observed in epithelial cells is due to the presence of a signal peptide, responsible for directing IL-1β to the apical membrane. However, further experimental investigation is required.

There has also been a growing appreciation and understanding of the role of the inflammasome in mediating IL-1β production in epithelial cell mediated immune responses, but the majority of this work has been carried out in human and murine intestinal tissue (31). However, the inflammasome has not been well characterized in cattle. Here nigericin induced IL-1β secretion within primary endometrial cell populations was investigated. In mice, loss of NLRP3 and caspase-1 completely abrogated IL-1β production (32–34). The contribution of both NLRP3 and caspase-1 to canonical inflammasome mediated IL-1β production in the human is well established (17, 35, 36). A role for NLRP3 and capase-1 in mediating IL-1β activation and release from bovine PBMCs has also been demonstrated (18). However, given the emerging evidence demonstrating discrepancies in inflammasome signaling across species, we first confirmed expression of the relevant inflammasome components within our cell populations, demonstrating the expression of these genes for the first time within the bovine and within endometrial cells. Additionally, treatment of endometrial cells with an inhibitor targeting the NLRP3 inflammasome receptor confirmed that IL-1β production in endometrial cells is NLRP3 dependent.

The data referred to above suggest a conserved role for caspase-1 in NLRP3 inflammasome formation. However, the absence of caspase-1 expression in our RNA-seq data sets from bovine uterine tissue suggests that a species-specific difference may exist in cattle. In contrast, caspase-4 was strongly differentially expressed between healthy and endometritic animals, suggesting it might play a role. This finding was confirmed by our qPCR analysis here. Examination of caspase-1 protein expression was precluded by the lack of availability of suitable specific antibodies for bovine caspase-1 or caspase-4 are not available. The FAM FLICA assay was used to detect active caspase-1/caspase-4 activity within our endometrial cells but was unable to discriminate between them. Reports on caspase-4 mediated IL-1β production are conflicting, with some reports suggesting it is required for mediating the cleavage of pro-caspase-1 into its active form (37). Alternatively, a report examining inflammasome activity in intestinal epithelial cells found that caspase-4 in humans (or its ortholog caspase-11 in mice) and not caspase-1 was required for inflammasome activity (38). A commercial inhibitor and siRNA targeting caspase-4 was used to conclusively establish the requirement for caspase-4 mediated IL-1β expression in the bovine endometrium. Given that we are unable to detect caspase-1 at the mRNA level and the ability of both the commercial caspase-4 inhibitor Z-LEVD-FMK and the caspase-4 specific siRNA to completely abrogate IL-1β production by both epithelial cells and stromal fibroblasts, we propose that caspase-4 mediates IL-1β cleavage into its active form within the endometrium through a non-canonical inflammasome pathway.

**Figure 9:**
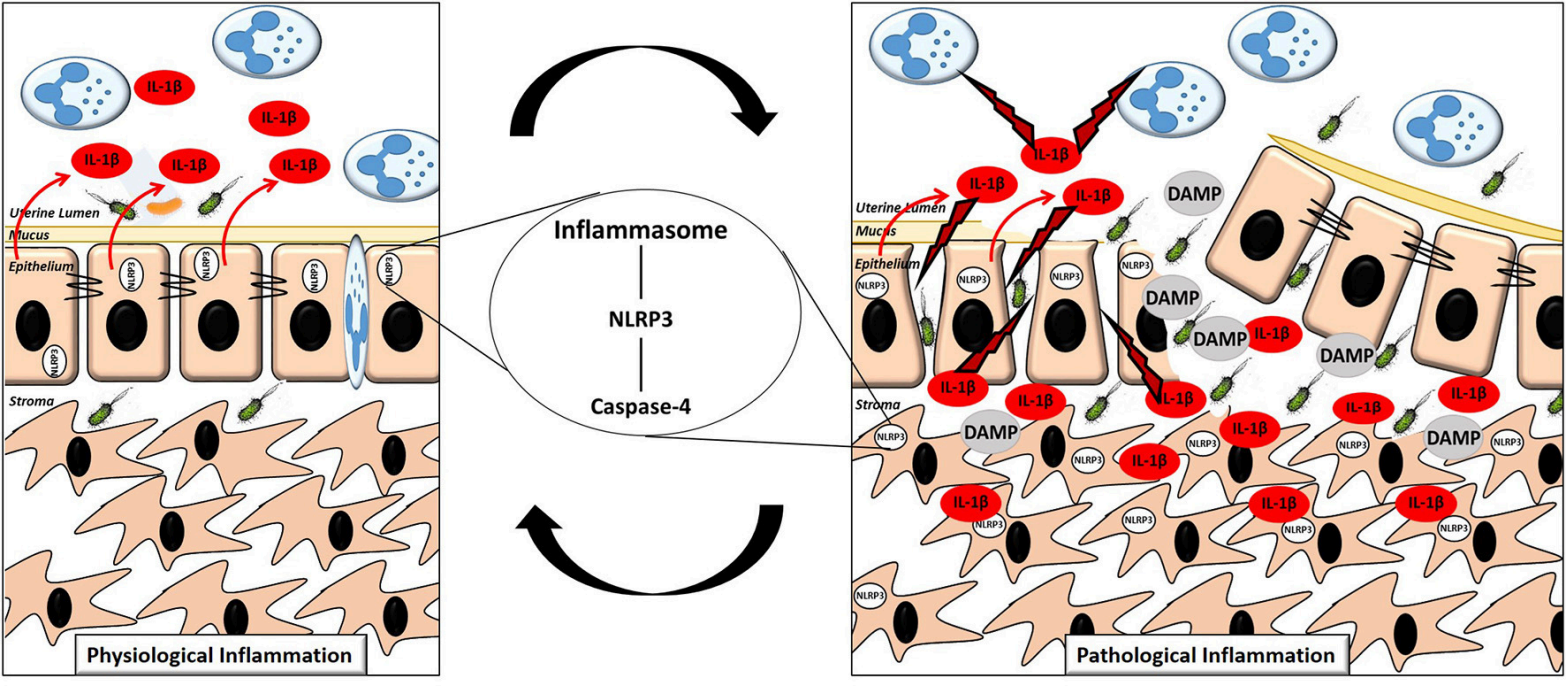
Proposed mechanism of the switch to pathological inflammation in endometritis. A physiological inflammatory response in the post-partum endometrium is characterized by a (mostly) intact epithelium, inflammasome activation in the epithelial cells with the majority of the subsequent release of the pro-inflammatory cytokine IL-1β directed into the uterine lumen. A pathological inflammatory immune response is characterized by a disrupted epithelium resulting in exposure of the stromal fibroblasts. Inflammasome activation subsequently occurs in the exposed stromal fibroblasts resulting in production of IL-1β and its release into both the lumen and surrounding endometrial tissue, propagating the pathological inflammation.

Inflammasome dependent production of IL-1β within the endometrium significantly enhances our appreciation for the molecular events surrounding the switch from physiological to pathological inflammation in the endometrium post-partum. We propose that during the early physiological inflammatory response in the 7 days following parturition, inflammasome activation occurs within the newly restored and intact epithelium, resulting in the release of IL-1β from the apical membrane into the uterine lumen. The IL-1β present in the lumen activates the immune cells which have been recruited, driving “extra-corporeal” protective immune activity (Figure 9). During a pathological inflammatory event, disruption to the epithelial barrier, as a consequence of dystocia during calving or a subsequent failure to re-establish the epithelial barrier post-partum, results in the underlying stromal fibroblasts becoming exposed for an extended period of time. Inflammasome activation occurs within the persistently exposed stromal fibroblasts resulting in excessive, undirected IL-1β production, causing a period of prolonged pathological inflammation which is detrimental to cow health and fertility (Figure 9).

Our work here has identified a role for the NLRP3 inflammasome complex in controlling IL-1β expression in the endometrium for the first time. In addition we have identified that uterine endometrial inflammasome activation is caspase-4 dependent, identifying a non-canonical inflammasome signaling pathway operating within the bovine endometrium. While the involvement of NLRP3 in the inflammasome complex is common across multiple cell types, a role for caspase-4 in mediating IL-1β production appears unique to cell populations found at mucosal sites like the endometrium. Understanding the detailed molecular regulatory mechanisms underpinning inflammasome activation within the endometrium is a crucial precursor to generating effective immunotherapeutic targets for the prevention of uterine disease in cattle.

## Methods

### Collection and Classification of Endometrial Samples

Endometrial biopsies were collected and classified as part of a previous study (4). Briefly. fifteen Holstein-Friesian cows, of mixed parity, within the same university dairy herd were sampled 7 and 21 days post-partum (DPP) in the morning after milking. A clinical examination and sample collection were conducted by a veterinarian. At each time-point, an endometrial biopsy was taken from the same post-gravid horn as previously described (39). Immediately after collection, the biopsy was fixed in 10% neutral-buffered formalin solution (Sigma Aldrich Poole, UK) for immunohistochemical assessment. Formalin fixed tissues were subsequently paraffin embedded.

Endometrial cytobrush samples were collected from mixed parity Holstein Frisian cattle at a commercial dairy farm. Sampling was carried out in the morning after milking at 7 and 21 days post-partum. Cytobrush collection was carried as previously described (40). Briefly, this involved trans-cervical insertion of a double guarded cytobrush into the uterus. Once the cervix was reached, the inner brush was pushed through the outer guard and rotated clockwise along the wall of the uterus three times to harvest the cells. The cytobrush was then re-inserted into the inner guard and removed, and stored in a labeled cryotube on dry ice. All tubes stored on dry ice were transferred to the −80°C freezer on return to the laboratory. A second inner cytobrush was inserted whilst the outer guard was still in place which enabled the collection of cells from the same position within the uterus. The second cytobrush was rolled anticlockwise on a glass microscope slide, and later stained with Diff-Quik (a modified Wright Giemsa stain) and used to classify the cows cytologically.

Once the cytology slides had been stained and dried, they were classified by observing the number of PMNs and epithelial cells present. The slides were graded blind without knowledge of sample ID or background. A total of 200 cells were counted at 400x magnification. The animals were classified in line with recent publications, using the cut off of 18% PMN at 21 DPP (40, 41). Those with ≤ 18% PMN at 21 DPP were classified as healthy, whilst cows with >18% PMN at 21 DPP were diagnosed with endometritis.

A vaginal mucus score was recorded for each cow at 7 and 21 DPP. Assessment of vaginal mucus was carried out using a gloved hand. A scoring system of 0–3 was used, as previously described (42). Briefly, a score of 0 or 1 was given to vaginal discharge that was clear or contained flecks of pus and the cow was classified as healthy. A score of 2 or 3 was given to samples with over 50% purulent or muco-purulent material present in the vaginal exudate and the cow was diagnosed with purulent vaginal discharge.

### Primary Cell Culture

Bovine female reproductive tracts were collected at a local abattoir within 15 min of slaughter. Collected tracts were identified as being free from disease and visible infection. Only tracts in the early luteal stage of oestrous (as identified by the presence of a stage 1 corpus luteum on one ovary) were used. Details of age and breed were also recorded at the time of collection.

The external surface of the tract was washed in 70% industrial methylated spirits (IMS) and the uterine horn ipsilateral to the corpus luteum was opened longitudinally with sterile scissors. The exposed endometrium was washed in PBS (Gibco, Paisley, UK) supplemented with 50 IU/ml penicillin, 50 IU/ml streptomycin (Gibco) and 2.5 g/ml amphotericin B (Sigma-Aldrich, Poole, UK). The endometrial surface was dissected using a sterile scissors and forceps. Dissection was performed at the abattoir with the tissue harvested into a 50 ml tube containing 20 ml of RPMI (Gibco) supplemented with antibiotics as above. Samples were transported to the laboratory at room temperature within 90 min of collection.

On arrival at the laboratory, tissue was washed twice in room temperature HBSS (Gibco, Madison, WI, UK) supplemented with antibiotics. The tissue was then chopped into fine pieces (~1–3 mm^3^ in size) using a sharp scissors and scalpels. The tissue was then incubated in HBSS with antibiotics at 37°C for 10 min in order to slowly return the tissue temperature to 37°C. HBSS was removed before the addition of 20 ml digestive solution (pre-warmed to 37°C). Digestive solution (100 ml) consisted of 375 BAEE units of trypsin-EDTA (Sigma-Aldrich), 50 mg collagenase II (Sigma-Aldrich), 100 mg bovine serum albumin (BSA) (Sigma-Aldrich), and 10 mg DNase I (Sigma-Aldrich) made up to 100 ml with HBSS and 0.2 micron sterile filtered (Sartorius, Göttingen, Germany). Samples incubated with digestive solution were placed in a 37°C shaking incubator at 150 rpm for 1 h.

The resulting mixture was filtered through a 70 μm nylon mesh cell strainer (Becton, Dickinson, Flintshire, UK) to remove any cell debris with the filtrate being collected in a tube containing HBSS with 10% FBS (Gibco). This solution was then passed through a 40 μm cell strainer (Pluristrainer, Pluiselect, Leipzig, Germany) to isolate the stromal cells. As the epithelial cells were too large to pass through the 40 μm cell strainer they remained on the strainer. The epithelial cells could then be isolated by turning the 40 μm cell strainer upside down and washing the epithelial cells into a clean 50 ml tube using 10 ml of HBSS supplemented with 10% FBS. Cells were then pelleted by centrifugation at 700 × g for 10 min. Red blood cells were lysed by re-suspending the pellet in 1 ml sterile water (Gibco) before addition of 4 ml of complete growth media. This consisted of RPMI 1640 supplemented with antibiotics as above, 10% FBS and 1X insulin, transferring, selenium supplement with ethanolamine (ITS-X) (Gibco).

Cells were pelleted as before and re-suspended in complete growth media. Cells were counted using a haemocytometer with trypan blue (Sigma-Aldrich, Poole, UK) staining to distinguish live from dead cells. Cell counts were adjusted to 3 × 10^5^ cells/ml and 8 × 10^5^ cells/ml for epithelial and stromal fibroblasts, respectively and 1 ml was seeded into 75 cm^2^ vented tissue culture flasks (Greiner Bio-One, Kremsmünster, Austria) with complete growth medium, which was stored in a 37°C incubator in 5% CO_2_. Cells were allowed to reach 85% confluency before plating, with media changing every 48 h. Visual examination of cell morphology under the light microscope was used to check purity of cultures in addition to characterization based on expression of differing cytoskeletal proteins.

### PBMC Isolation and Culture

Bovine peripheral blood mononuclear cells (PBMCs) were isolated from whole blood samples collected in 9 ml vacutainers containing Heparin anticoagulant (Greiner Bio-One, Kremsmünster, Austria). Whole blood was diluted 1:1 with HBSS and mixed gently before 25 ml was layered onto 15 ml of sterile Ficoll-paque density gradient medium (Fisher Scientific, New Hampshire, US) in a 50 ml tube. The samples were then centrifuged at 800 × g for 25 min with the centrifuge break turned off. The mononuclear cells were carefully removed with a pastette into a new 50 ml tube and made up to 10 ml with wash buffer (HBSS and 5% FBS). Cells were pelleted by centrifugation at 300 × g for 5 min. Any red blood cells carried over during the separation process were lysed by re-suspending the cells in red blood cell lysis buffer (Sigma-Aldrich). The cell pellet was then re-suspended in complete RPMI media (containing 10% FBS and 1% Pen-Strep) and cells were counted using a haemocytometer and trypan blue staining. Cell were then seeded at appropriate densities in tissue culture plates containing media. The cells were incubated at 37°C with 5% CO_2_.

### Polarized Epithelial Cell Culture

Polarized epithelial cell cultures were prepared by seeding 1.5 × 10^5^ cells on each hanging cell culture insert, which had previously been coated with 50 μl of matrigel (Corning, New York, USA) diluted 1:8 in RPMI 1640 and left at room temperature for 1 h before the remaining matrigel was aspirated. Inserts were placed in 24-well plates, with 300 and 800 μl of culture medium in the apical and basolateral compartment, respectively. Confluence of the epithelial layer was determined by a FITC-dextran permeability assay, were 0% permeability compared to the no cell control was indicative of cells reaching confluency, and by examining the TER of the epithelial barrier using EVOM2 epithelial voltohmmeter (World Precision Instruments) where high resistance (>1,000 Ωcm^2^) was indicative of confluency and successful polarization. The polarized epithelial cells were treated after ~14 days of culture.

### siRNA Treatments

siRNA duplexes targeting bovine caspase-4 (Sense: GGAAGAACCAGAUGUGUUAUU; Antisense: UAACACAUCUGGUUCUUCCUU) were designed using Dharmacon's siDESIGN Center. For control, a non-targeting siRNA #1 (scramble) was used. Cells were seeded in a 24 well plate (stromal fibroblasts) or on hanging inserts (epithelial cells) cells to be 60–80% confluent at the time of transfection. Lipofectamine (RNAiMax, Invitrogen) was diluted by adding 3 μl lipofectamine to 50 μl Opti-mem medium (Gibco). siRNA targeting caspase-4 or non-targeting siRNA (scramble) (20 μM stock) was diluted 1:50 in Opti-mem medium. The diluted siRNA was added to the diluted lipofectamine (1:1 ratio) and incubated for 5 min at room temperature. The siRNA complex was then added dropwise to cells (100 ul of siRNA complex added to 1 ml complete RPMI media) to give a final concentration of 5 pmol siRNA in each well. Cells were incubated for 48 h at 37°C before the media was replaced and the transfected cells were stimulated with the inflammasome activating agents LPS and nigericin for 6 h. Following stimulation supernatants were harvested for IL-1β analysis by ELISA and cells were harvested in TRIzol for caspase-4 mRNA expression analysis.

### Cell Treatments

For time-point stimulations of epithelial cells and stromal fibroblasts, cells were plated at a density of 1.5 × 10^5^ cells/ml in a 24 well plate and left to rest for 24 h before stimulation. Following the 24 h rest period, media was removed and replaced with control media or media containing 2 μg/ml LPS (Enzo Life Sciences, Exeter, UK) for 24, 12, 6, or 3 h before addition of 10 μM nigericin for 1 h.

For inhibitor experiments, cells were plated at a density of 1.5 × 10^5^ cells/ml in a 24 well plate and left to rest for 24 h before treatment. Cells were then treated with increasing concentrations of MCC950 (1–100 nM) (a kind gift from Prof. Luke O'Neill), Z-VAD-FMK (1–100 nM) (Invivogen) or Z-LEVD-FMK (0.02–2 μM) (Enzo Life Sciences) for 1 h before the addition of LPS for 6 h followed by addition of nigericin for 1 h.

Following stimulation cells were either pelleted and lysed in RIPA buffer supplemented with protease inhibitors for protein analysis or lysed in TRIzol for mRNA extraction and stored at −80°C. Supernatants were harvested and stored at −20°C for further analysis.

### qPCR

Total RNA was extracted using TRIzol reagent according to manufacturer's instructions. RNA quantity was calculated using ND-1000 NanoDrop spectrophotometer (Thermo Fisher Scientific). One microgram of RNA was reverse transcribed into cDNA using the OmniScript kit (Qiagen, Crawley, UK) with Oligo (dT) primers (Qiagen) according to the manufacturer's instructions. cDNA solutions were diluted 1:10 before qPCR analysis.

qPCR was performed using primers detailed in Supplemental Table 1. Primers were designed using the Primer BLAST software to be intron spanning where possible. Optimal primer concentrations were assessed by titrating different final concentrations and dissociation curves were examined for the presence of a single product.

Quantitative PCR was run using the PowerUp SYBR Green Master Mix (Thermo Fisher Scientific) on a StepOnePlus Real-Time PCR System (Thermo Fisher Scientific) using the following parameters: 95°C for 20 s, followed by 40 cycles of 95°C for 3 s and 60°C for 30 s and a final amplicon dissociation step at 95°C for 15 s, 60°C for 1 min and 95°C for 15 s. *H3F3A* was found to be the most stably expressed reference gene from a panel of reference genes tested using GeNorm software and was subsequently used to generate normalized relative expression values (43).

### Western Blotting

Epithelial or stromal fibroblast cells (1 × 10^6^ cells/ml) were harvested in RIPA buffer supplemented with the protease inhibitors leupeptin (2 μg/ml), aprotonin (2 μg/ml), sodium orthovanadate (4 μg/ml), and phenylmethylsulphonyl fluoride (100 μg/ml). Protein quantification was performed using BCA Protein Assay Reagents (Pierce Biotech) according to the manufacturer's instructions. A standard curve was generated each time with BSA standards and the protein concentration of each sample was calculated from this. From these values, protein samples were normalized relative to the sample of lowest concentration in 20 μl dilutions. Loading buffer (4X Tris-glycine SDS β-mercapthenol) was added to each sample (5 μl) and boiled at 95°C for 5 min.

Prior to SDS-PAGE a 15% running gel, topped with a 4% stacking gel, were prepared. The gels were loaded into the running tank and 1X Running buffer (0.025 M Tris, 0.192 M glycine, 0.1% SDS) was added and poured into the upper and lower reservoirs of the apparatus. The samples and 3 μl molecular marker was used per gel. The gel was run at 110 V for 45 min. After the run, the stacking gel was removed and the running gel retained for transfer to PVDF membranes.

Transfer buffer (0.048 M Tris, 0.039 M glycine, 20% methanol, 0.00375% SDS), PVDF membrane (0.2 μm pore size) and blotting pads were prepared. The transfer membrane were cut to correct size and activated by immersion in methanol for 30 s and then in dH_2_0 for 2 min. The membrane and blotting pads were then soaked in transfer membrane for 5 min. Once the gel had ran, it was removed from the gel apparatus and placed in transfer buffer for 5 min. The gel was then transferred to the transfer machine where it was sandwiched between blotting pads and a transfer membrane. The transfer machine was connected to a voltmeter and run at 10 V (250 mA) for 20 min.

Once the membrane was removed from the transfer machine, it was blocked to prevent non-specific binding of antibody with 5% milk in PBS tween (PBS-T) for 1 h. After blocking, the primary antibody was added and the blot was left rocking in the cold room overnight. Primary antibody was diluted to a concentration of 1:1,500 in 30 mls of PBS-T. Before adding the secondary antibody, the membrane was washed for 5 min three times in PBS-T. Secondary antibodies were applied (1:5,000 dilution in 5% milk) and the blot was left on the rocker for 1 h at room temperature. The blot was then washed again for 5 min three times, before being incubated in ECL (Biorad) and visualized on the Biorad ChemiDoc MP System.

### ELISA

Quantification of IL-1β was performed using the Bovine IL-1β ELISA kit according to the manufacturer's instructions (Thermo Scientific). Samples were assayed in duplicate and measured at 450 nm with wavelength correction at 620 nm. Quantification of IL-8 was performed according to a previously reported protocol (44). Supernatants from endometrial cell stimulations were used neat or diluted as required and cytobrush samples were diluted to a concentration of 1 mg/ml. Values were determined using four-parameter logistic regression of the standard curve.

### Immunohistochemistry

Paraffin embedded endometrial biopsies were sectioned at five micrometers, incubated at 70°C overnight and dewaxed by three immersions in histoclear for 10 min each before rehydration with 100, 70, and 50% IMS and dH_2_O for 5 min each. Heat induced antigen retrieval using a microwavable pressure cooker was performed in sodium citrate buffer (pH 6), sections were then incubated in PBS for 5 min before incubation in casein (diluted 1 in 5 with PBS) for 20 min to block any non-specific staining. Rabbit polyclonal anti-IL-1β (1:75) or anti-CD45 (1:200) was applied overnight at 4°C in a humid chamber. Negative control sections were incubated with rabbit IgG. The following day the sections were washed twice in PBS before a peroxidase block (30% H_2_O_2_ diluted 1:10 in PBS) was applied for 7 min, followed by one wash in PBS and incubation with secondary antibody attached to a peroxidase-conjugated polymer (Dako) for 30 min at room temperature. Sections were washed twice more before the substrate chromagen diaminobenzidin (DAB) (Dako) was applied for a maximum of 10 min. The reaction was then stopped with dH_2_O and washed a further two times in dH_2_O before the sections were counterstained with 0.2 μm filtered haematoxylin, dehydrated, and mounted with mounting medium. Slides were visualized using the Aperio ScanScope Imager.

### FLICA™ Assay

The FLICA™ Assay Kit (Immunochemistry Technologies, Minneapolis, USA) was used to examine caspase-1/4 expression. Cells were seeded at a density of 0.5 × 10^6^ and allowed to rest overnight before stimulation with LPS (2 μg/ml) for 3 h followed by nigericin (10 μM) for 1 h. Following stimulation PBMCs were re-suspended and endometrial cells lifted with accutase treatment for 5 min before centrifugation at 300 × g for 5 min to pellet the cells. Supernatants were discarded and cells were re-suspended in FACs buffer (PBS with 5% FBS and 0.1% sodium azide) and incubated for 30 min at 37°C with the caspase probe (FAM-YVAD-FMK). Following this, cells were centrifuged and washed 3 times in FACs buffer. The live/dead stain 7AAD was added and cells were subsequently analyzed by flow cytometry using a BD Accuri C6 flow cytometer (BD Biosciences, New Jersey, USA). Cells were analyzed on a FSC-A vs. FSC-H plot to exclude doublets followed by FSC-A vs. SSC-A to omit debris. Dead cells were excluded using the 7AAD live/dead stain before gating on FLICA^+^ cells. Data was analyzed using FlowJo software (Version 7.6.5, Tree Star, Ashland, OR, USA).

### Cell Viability Assays

Epithelial cell viability was examined by measuring the TER, which is indicative of epithelial barrier integrity. TER was measured using a EVOM2 epithelial voltohmmeter (World Precision Instruments) where high resistance (>1,000 Ωcm^2^) indicated an intact epithelium and by default, viable cells. Stromal fibroblast viability was quantified using a Cell Titer Blue Viability Assay (Promega, Wisconsin, USA) according to manufacturer's instructions. Briefly, cells were seeded at cells/ml in a 96 well plate. Treatments were performed and following this, media was removed and replaced with 100 μl fresh media per well to which 10 μl of Cell Titer blue dye was added. Plates were returned to the incubator for 3 h before fluorescence was recorded at 560/590 nm.

### Statistical Analysis

For the analysis of 2 groups where the data was independent a Wilcoxon rank sum test (Mann Whitney *U*-test) was used. For the analysis of more than 2 groups a Kruskal-Wallis test was used with Dunns multiple comparison *post-hoc* test. A *P* < 0.05 was considered statistically significant. Statistical analysis was performed using GraphPad Prism 7 software.

## Ethics Statement

All procedures described were conducted under ethical approval and experimental license from the Irish Health Products Regulatory Authority in accordance with the Cruelty to Animals Act 1876 and in agreement with the European Union (Protection of Animals Used for Scientific Purposes) regulations 2012 (S.I. No. 543 of 2012).

## Author Contributions

PK designed and conducted experiments, analyzed data and wrote the paper. KM and CO co-directed the study and wrote the paper.

### Conflict of Interest Statement

The authors declare that the research was conducted in the absence of any commercial or financial relationships that could be construed as a potential conflict of interest.
