# Supplementary material for: Non-canonical Inflammasome-Mediated IL-1β Production by Primary Endometrial Epithelial and Stromal Fibroblast Cells Is NLRP3 and Caspase-4 Dependent
Source: Front Immunol. 2019 Feb 5;10:102. doi: 10.3389/fimmu.2019.00102 (PMC6371858; doi:10.3389/fimmu.2019.00102)
Supplement: Supplementary file 1 [file Data_Sheet_1.docx]

***Supplementary Information***

**Non-canonical inflammasome mediated IL-1β production by primary endometrial epithelial and stromal fibroblast cells is NLRP3 and caspase-4 dependent.**

Paul Kelly^1, 2^, Kieran G. Meade^2¥^ and Cliona O’Farrelly^1, 3¥*^

^1^Comparative Immunology Group, School of Biochemistry and Immunology, Trinity Biomedical Sciences Institute, Trinity College Dublin, Dublin, Ireland.

^2^Animal and Bioscience Research Department, Animal and Grassland Research and Innovation Centre, Teagasc, Grange, Ireland.

^3^School of Medicine, Trinity College Dublin, Dublin, Ireland.

^¥^These authors contributed equally to the direction of this work

*****Correspondence: Cliona O’Farrelly ([cliona.ofarrelly@tcd.ie](mailto:cliona.ofarrelly@tcd.ie))

**Supplemental Figure 1. IL-1β is present in endometrial epithelial cell lysates.**

**
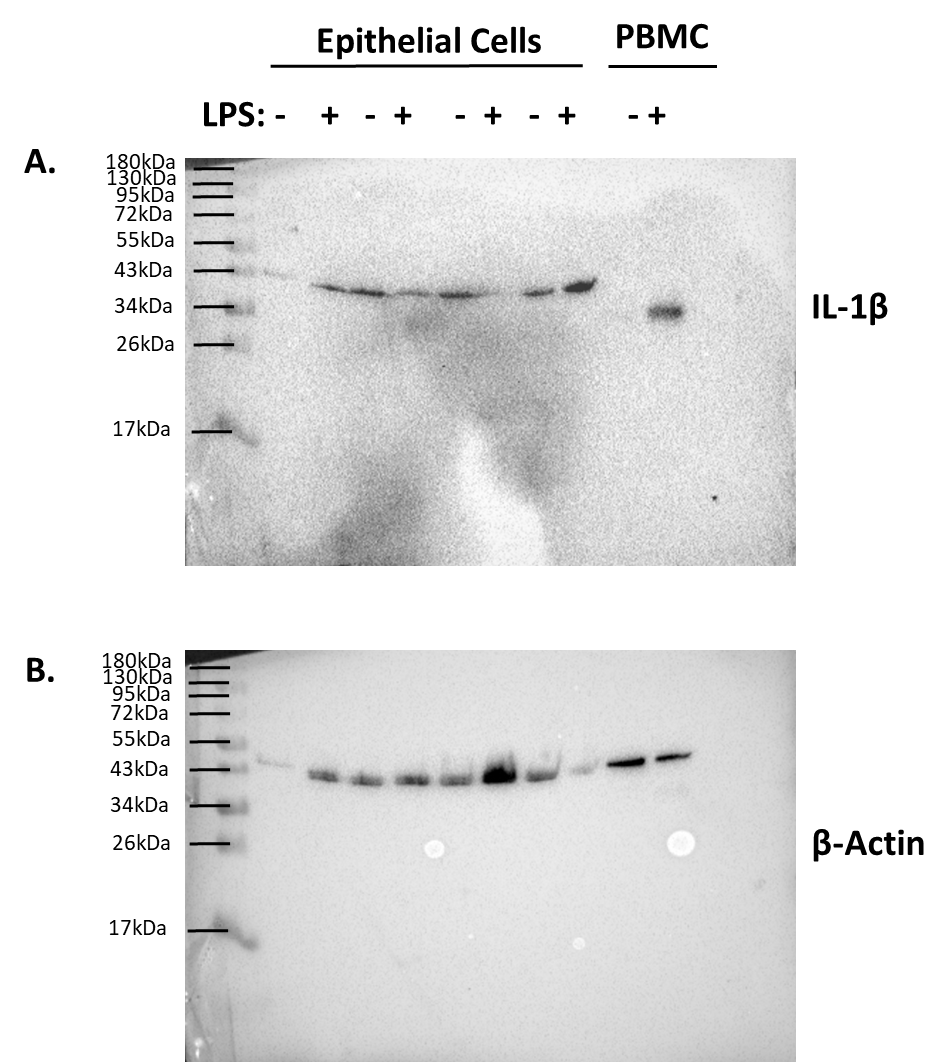
**

**Supplemental figure 1. IL-1β is present in endometrial epithelial cell lysates.**

**(A.).** Pro-IL-1β and **(B.).** β-Actin protein expression was examined in epithelial cells by western blotting. PBMC lysates are included as a positive control.

**Supplemental figure 2. IL-1β is present in endometrial stromal cell lysates.**

**
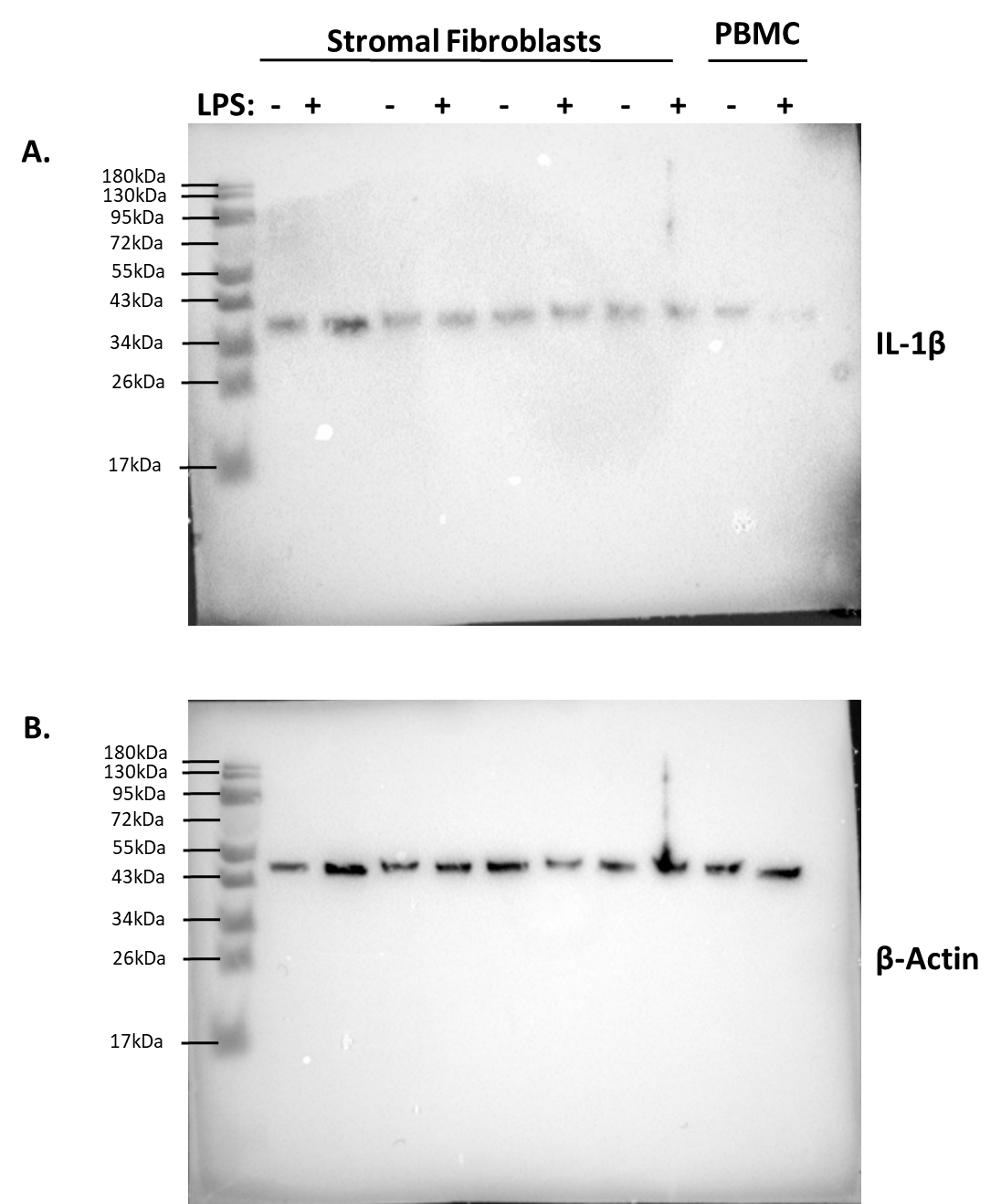
**

**Supplemental figure 2. IL-1β is present in endometrial stromal fibroblast lysates.**

**(A.).** Pro-IL-1β and **(B.).** β-Actin protein expression was examined in stromal fibroblasts by western blotting. PBMC lysates are included as a positive control.

**Supplemental figure 3. Cell viability and IL-8 cytokine production are unaffected by MCC950 and Z-VAD-FMK.**

**
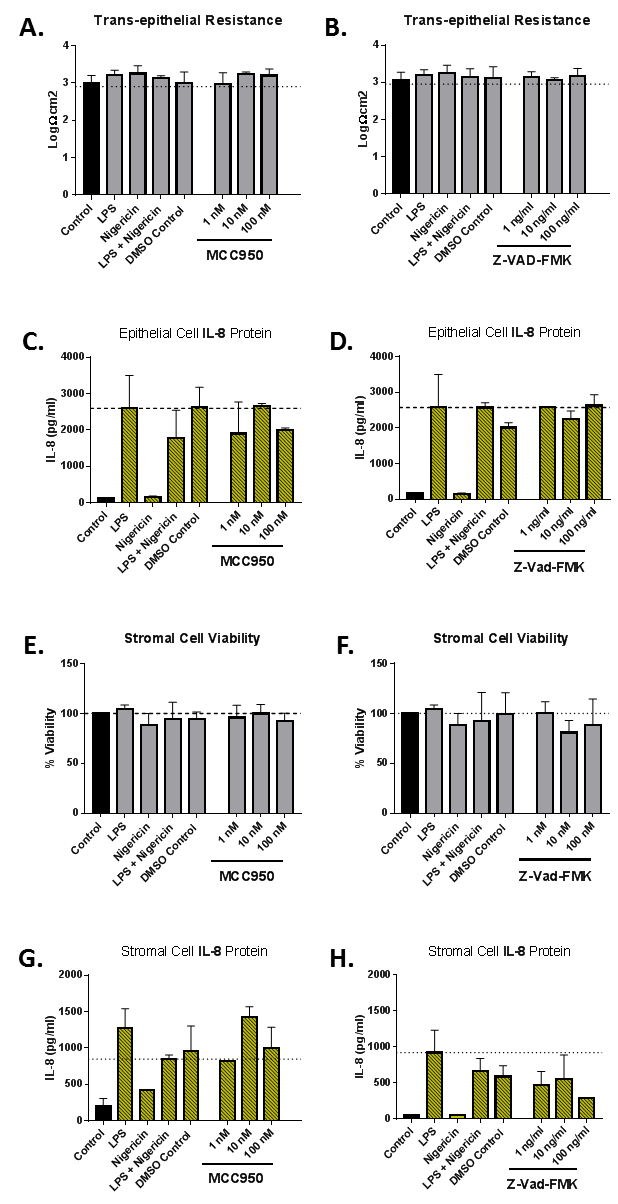
**

**Supplemental figure 3. Cell viability and IL-8 cytokine production are unaffected by MCC950 and Z-VAD-FMK.**

**(A.-B.).** The effect of the inhibitors MCC950 and Z-VAD-FMK on epithelial cell viability was examined by analysing trans-epithelial resistance (TER), a measurement of epithelial barrier integrity (*n*=5). **(C.-D.).** IL-8 cytokine production by epithelial cells following treatment with MCC950 or Z-VAD-FMK was measured by ELISA (*n*=5). **(E.-F.).** The effect of the inhibitors MCC950 and Z-VAD-FMK on stromal fibroblast viability was examined by the Cell Titer Blue™ viability assay (*n*=5). **(G.-H.).** IL-8 cytokine production by stromal fibroblasts following treatment with MCC950 or Z-VAD-FMK was measured by ELISA (*n*=5).

**Supplemental figure 4. Cell viability and IL-8 cytokine production are unaffected by Z-LEVD-FMK and siRNA targeting caspase-4.**


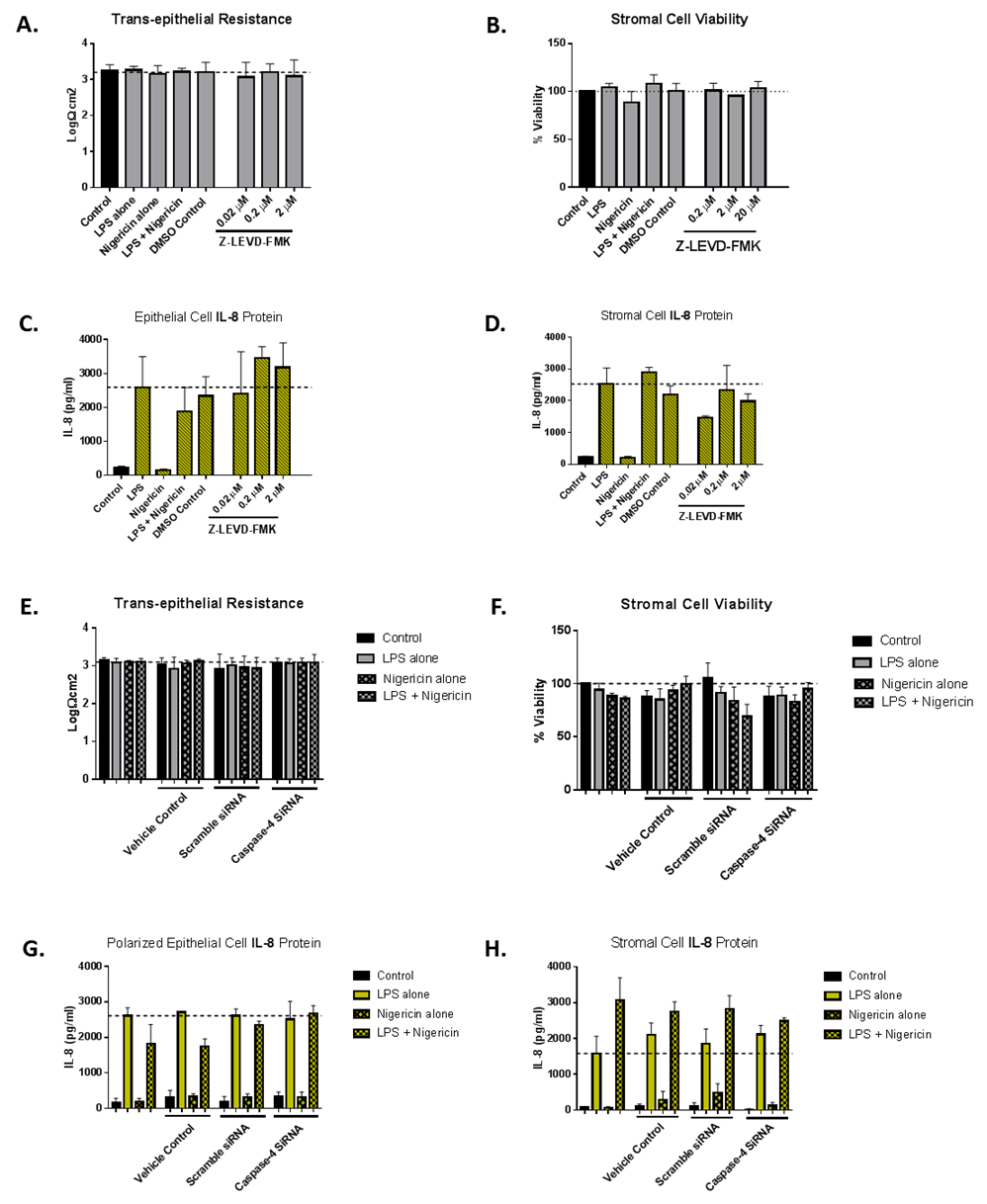


**Supplemental figure 4. Cell viability and IL-8 cytokine production are unaffected by Z-LEVD-FMK and siRNA targeting caspase-4.**

**(A-B).** The effect of the inhibitors Z-LEVD-FMK on epithelial cell or stromal fibroblast viability was examined by analysing trans-epithelial resistance (TER) or performing a Cell Titer Blue™ viability assay respectively (*n*=5). **(C-D).** IL-8 cytokine production by epithelial cells or stromal fibroblasts following treatment with Z-LEVD-FMK was measured by ELISA (*n*=5). **(E-F).** The effect of treatment with siRNA targeting caspase-4 on epithelial cell or stromal fibroblast viability was examined by analysing trans-epithelial resistance (TER) or performing a Cell Titer Blue™ viability assay respectively (*n*=5). **(G-H).** IL-8 cytokine production by epithelial cells or stromal fibroblasts following treatment with siRNA targeting caspase-4 was measured by ELISA (*n*=5).

**Supplemental table 1. Details of primers used for qPCR analysis.**

| **Gene** | **Forward Primer** | **Reverse Primer** | **Product Size** | **Concentration** |
| --- | --- | --- | --- | --- |
| IL1B | AACGTCCTCCGACGAGTTTC | GCTCATGCAGAACACCACTTC | 163bp | 500nM |
| NLRP3 | CTTTCTGGACTCTGACCGGG | CTCCCATTCTGGCTCTTCCC | 312bp | 300nM |
| NLRP1 | TCATTCTCCCACTCGCCAAG | TATCTCTGTCTGCTTCCTGTGC | 371bp | 300nM |
| NLRC4 | CAGCTGAACCCTGTGACCTT | GCAGCCTGTCAATTAGTTGCT | 180bp | 300nm |
| IFI16 | GGAAGTCGTGGTTTATGGACAGCG | CCTTGGTGACCTTGATGAAACTATGAAT | 146bp | 300nM |
| NEK-7 | ACCTCTCCGCTTCCTGAGTTC | AGCCCATATCCGGTCGTAAG | 126bp | 300nM |
| ASC | ATGAAGCTGCTTTCAGTGCC | ACAAAGTGCAGTCCTGGCTTG | 272bp | 500nM |
| Gasdermin D | CCATTGGGAGCATGGCCTC | GCGGTCTCCAGAATCGTGAA | 165bp | 300nM |
| Caspase-1 | AATAAATGGCTTGCTGGATGAG | CCTCCTGGTCCTGAAGATGC | 327bp | 500nM |
| Caspase-4 | TATAAAAGCTCCTGAGGAAACT | TTCCACAGTGTAGCCAAGAC | 268bp | 500nM |
| H3F3A | CATGGCTCGTACAAAGCAGA | ACCAGGCCTGTAACGATGAG | 136bp | 100nM |
